# Echocardiography for adult patients supported with extracorporeal membrane oxygenation

**DOI:** 10.1186/s13054-015-1042-2

**Published:** 2015-10-02

**Authors:** Ghislaine Douflé, Andrew Roscoe, Filio Billia, Eddy Fan

**Affiliations:** Interdepartmental Division of Critical Care Medicine, University of Toronto, Toronto, ON M5G 2N2 Canada; Extracorporeal Life Support (ECLS) Program, Toronto General Hospital, Toronto, ON M5G 2N2 Canada; Department of Anaesthesia & ICU, Papworth Hospital, Cambridge, CB23 3RE UK; Peter Munk Cardiac Centre, University Health Network, Toronto, ON M5G 2N2 Canada

## Abstract

Venoarterial (VA) and venovenous (VV) extracorporeal membrane oxygenation (ECMO) support is increasingly being used in recent years in the adult population. Owing to the underlying disease precipitating severe respiratory or cardiac failure, echocardiography plays an important role in the management of these patients. Nevertheless, there are currently no guidelines on the use of echocardiography in the setting of ECMO support. This review describes the current state of application of echocardiography for patients supported with both VA and VV ECMO.

**Electronic supplementary material:**

The online version of this article (doi:10.1186/s13054-015-1042-2) contains supplementary material, which is available to authorized users.

## Introduction

The use of extracorporeal membrane oxygenation (ECMO) in adults has grown over recent years, particularly venovenous (VV) ECMO for respiratory failure [1]. A recently published position paper on the management of ECMO recommends that an echocardiography-trained physician should be part of the team caring for patients on ECMO [2]. However, the published literature on the role of echocardiography in ECMO remains scarce and is mainly confined to the pediatric population [3–8]. In this article, we review the current state of knowledge and application of echocardiography in adult patients supported with ECMO. A brief overview of the various modes of ECMO will be covered to provide the basis for understanding the role of echocardiography in this patient population, but the authors refer the readers to previous publications for more detailed reviews of ECMO [9–11].

## Overview of ECMO

**Fig. 1:**
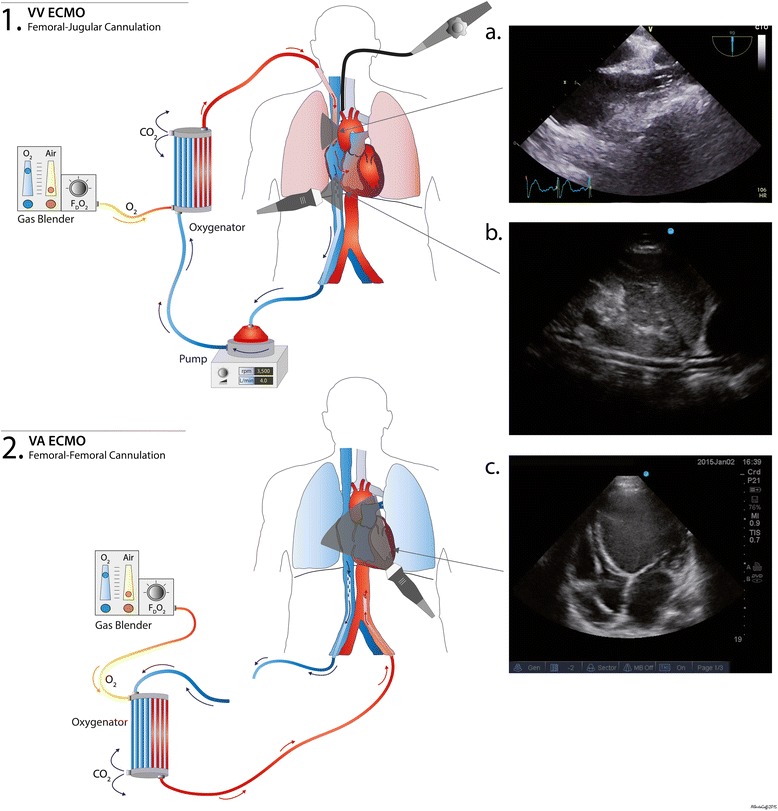
Venovenous (VV) and venoarterial (VA) extracorporeal membrane oxygenation (ECMO) configurations and corresponding echocardiographic views. This diagram shows the most common ECMO configurations in our center. (1) Bicannulation VV ECMO (femoro-jugular cannulation) with the drainage cannula in the femoral vein and the reinjection in the superior vena cava (SVC), via the jugular vein. **a** Mid-esophageal view showing the SVC and reinjection cannula within it (transesophageal echocardiography). **b** Inferior vena cava (IVC) subcostal view. The drainage cannula is visualized within the IVC in long axis (transthoracic echocardiography). (2) Femoro-femoral VA ECMO cannulation. The drainage cannula is located in the IVC and the reinjection cannula is in the iliac artery/distal descending aorta. **c** Transthoracic apical four-chamber of a patient with a dilated cardiomyopathy. The cannulae are not visualized on this view but note the presence of an automatic implantable cardioverter defibrillator within the right ventricle

The two main configurations of ECMO currently used are VV ECMO, for respiratory support, and venoarterial (VA) ECMO, for cardiorespiratory support (Fig. 1). VV ECMO does not directly provide cardiac support; however, resolution of severe hypoxemia and hypercapnia will decrease the pulmonary vascular resistance, reducing the right ventricle (RV) afterload and improving RV function [12]. This should augment left ventricle (LV) filling. In addition, the institution of VV ECMO usually results in reduction of airway pressures, which also decreases RV afterload. In patients with cardiorespiratory compromise, VA ECMO is the modality of choice and provides full cardiopulmonary support.

## Pre-ECMO assessment

Prior to initiating ECMO support, a comprehensive echocardiographic examination should be undertaken, as permitted by the patient’s hemodynamic condition (Table 1). Cardiovascular collapse prompting the urgent institution of ECMO will obviously preclude the performance of a complete echocardiographic study. In extreme situations, during extracorporeal cardiopulmonary resuscitation for refractory cardiac arrest, only anatomical aspects of cardiac chambers and valves can be evaluated, but echocardiography may rapidly diagnose a readily reversible etiology, such as cardiac tamponade [13].

**Table 1:** Parameters to be assessed prior to extracorporeal membrane oxygenation initiation

Left ventricle	
Morphology	Size, wall thickness
Systolic function	Ejection fraction (Simpson’s method) or FAC
	Wall motion abnormalities
	S wave at mitral annulus
	Velocity time integral in LVOT
Diastolic function	E/A ratio (trans-mitral flow)
	E/e’ ratio at mitral annulus
Left atrium	Size and volume
Valvular assessment	Diagnosis and quantification of potential aortic/mitral regurgitation/stenosis
Right ventricle	
Morphology	RV end diastolic area/LV end diastolic area
	Triangular shape versus rounded shape of apex
	RV wall thickness
	McConnell’s sign
Systolic function	TAPSE, tissue Doppler at tricuspid annulus, S wave
	Fractional area of change
	Pulse wave Doppler through pulmonary valve (acceleration time/biphasic pattern)
Diastolic function	E/A trans-tricuspid flow
Interventricular septum	Presence of paradoxical septum
	Eccentricity index
Tricuspid regurgitation	Estimation of right ventricular systolic pressure
Valvular pathology	
Right atrium	Size and volume
Other	
Patent foramen ovale	Color flow Doppler ± bubble study
IVC/SVC (if TEE)	Size and respiratory variation
Right atrium	Dilated coronary sinus
	Chiari network
Vascular	Thrombosis/stenosis/aortic dissection/severe atheroma

*E/A* early diastolic peak velocity/diastolic ventricular filling with atrial contraction, *E/e’* early diastolic peak velocity/early diastolic tissue Doppler velocity, *FAC* fractional area change, *IVC* inferior vena cava, *LV* left ventricle, *LVOT* left ventricular outflow tract, *RV* right ventricle, *SVC* superior vena cava, *TAPSE* tricuspid annular plane systolic excursion, *TEE* transesophageal echocardiography

The choice between VV and VA ECMO support will depend on the underlying etiology of the patient’s decompensation. Whilst the incidence of RV dysfunction has been dramatically reduced in patients with acute respiratory distress syndrome (ARDS) by the implementation of protective lung ventilation, it still remains as high as 25 % [14, 15]. Even with predominant respiratory failure, the choice of ECMO modality that would best benefit the patient is not always straightforward. Institution of VV ECMO leads to resolution of hypoxemia and hypercapnia, with lower airway pressures required, resulting in decreased pulmonary vascular resistance. This may reverse the hemodynamic instability associated with RV dysfunction. However, causes of RV failure may not be immediately reversible, and it may be difficult to ascertain what proportion of the hemodynamic instability can be ascribed to the underlying metabolic disturbances. The presence of significant concomitant LV dysfunction warrants the use of VA ECMO, and echocardiography plays a crucial role in assessing the degree of residual LV contractility.

Echocardiographic features of RV dysfunction include RV chamber dilatation, flattening of the interventricular septum, creating a D-shaped LV with an eccentricity index greater than 1; right atrium (RA) and tricuspid valve (TV) annular dilation, with significant tricuspid regurgitation (TR); and RV contribution to the apex of the heart, typically visualized in the transthoracic echocardiography (TTE) or transesophageal echocardiography (TEE) four-chamber view. Objective measurements of RV systolic function include the tricuspid annular plane systolic excursion; the tricuspid annular systolic peak velocity (S’), using tissue Doppler imaging; and fractional area change. McConnell’s sign, severe basal and mid RV hypokinesia with apical hyperkinesia, is indicative of acute onset pulmonary hypertension [16, 17].

The RV systolic pressure can be estimated using the simplified Bernoulli equation, by measuring the peak velocity of the TR jet. Increased pulmonary artery (PA) pressure can also be estimated by pulse wave Doppler interrogation of the PA systolic flow. The presence of a biphasic waveform and a PA acceleration time (time from the onset of systole to time to peak pressure) shorter than 100 ms are indicative of increased pulmonary pressure [18]. These numbers will serve as a reference as the TR is not considered to be accurate once the patient is on ECMO support. Indeed, drainage and reinjection of blood from and into the RA make it impossible to accurately quantify TR. The drainage of blood from the RA will likely alter the pressure gradient between RV and RA, precluding an accurate assessment of the RV systolic pressure. However, it is important to bear in mind that in acute RV failure, the PA pressure might not be elevated if the RV cannot generate a sufficient pressure in the setting of acutely increased afterload.

Although imperfect, the objective assessment of the LV includes the measurement of its size along with global and regional function. The presence of thin walls usually indicates a chronic process. Similarly, a dilated left atrium (LA) will be in keeping with chronically elevated left atrial and ventricular diastolic pressures. The global function can be assessed by the modified Simpson’s method, to give an estimate of ejection fraction (EF) [16]. The consideration for VA ECMO implies severely decreased systolic function, with an EF <20 %, with or without wall motion abnormalities.

Pre-extracorporeal life support, a TTE or TEE study is useful to detect certain important pathologies. The initiation of VA ECMO increases the LV afterload and the severity of any pre-existing aortic regurgitation will worsen, leading to increasing LV dilatation, pulmonary edema and risk of subendocardial ischemia secondary to increased myocardial oxygen consumption and wall stress [19–22]. The presence of significant mitral regurgitation may be associated with increased pulmonary edema. It is important to determine the etiology of any significant mitral regurgitation to exclude pathology that may be amenable to surgical intervention.

Similarly, the presence of TV pathologies should be assessed. Although extremely rare in adults, presence of tricuspid stenosis will compromise the flow of the oxygenated blood from the RA to the RV. Acute severe TR with ensuing increased right atrial pressures and right to left shunt might manifest with severe hypoxemia, by opening a patent foramen ovale (PFO). The presence of intracardiac shunts, such as an atrial septal defect or a PFO, may not impact oxygenation directly whilst the patient is supported with ECMO; it may, however, have consequences when weaning from ECMO, as an increase in right heart pressures during the weaning process could precipitate a right-to-left shunt through an atrial septal defect or PFO.

Examination of the aorta is essential, as aortic dissection may be the underlying cause of hemodynamic instability and urgent surgical repair may be warranted. Additionally, aortic dissection is a relative contraindication to VA ECMO.

The presence and size of any pericardial fluid must be determined to enable differentiation between a pre-existing effusion and an iatrogenic collection due to cannulation.

Normal variants and embryological remnants might be found and should be reported, especially the presence of prominent Chiari network or the presence of dilated coronary sinus with or without a persistent left superior vena cava (SVC). A prominent Chiari network might hinder proper advancement of the cannulae and expose the patient to a higher risk of thrombosis. A dilated coronary sinus can potentially be easier to be cannulated: if accidentally cannulated, in the presence of a persistent left SVC, oxygenation can be compromised, with oxygenated blood being reinjected toward the left arm instead of the RA [23].

## Cannulation

There are currently no recommendations on which imaging modality is superior to guide ECMO cannulation. Insertion may be guided by fluoroscopy, TTE or TEE. Each of these modalities has its advantages and disadvantages. Echocardiography has the ability to determine the exact position of the cannula, although one should be cognizant of the presence of artifacts that might be misleading. It also allows for prompt diagnosis of any cannulation complications, such as pericardial effusion or aortic dissection.

Prior to cannulation, a thorough examination of vascular anatomy will assist in determining any potential barriers to cannulation. It allows identification of thrombus, vessel stenosis, aneurysms or severe atheromatous disease, thus assisting cannulation. The size of the venous drainage cannula is a major determinant of blood flow in the ECMO circuit; therefore, an attempt is made to insert the largest cannula possible. Measuring the diameter of the vessels may aid the choice of cannula size [24, 25]. The arterial inflow cannula is typically smaller in size, but in patients with small femoral arteries a distal perfusion line should be considered to ensure adequate blood flow to the lower limb [26, 27].

In VV ECMO, the percutaneous approach is typically the method of choice. The blood is drained from the venous system and returned to the patient’s venous system after oxygenation and carbon dioxide (CO_2_) removal. This may be achieved with two cannulae, typically inserted in the femoral vein, and advanced into the inferior vena cava (IVC), and the internal jugular vein, advanced into the SVC. Alternatively, both femoral veins are cannulated and the reinjection cannula is advanced into the RA. The other option is a single, dual-lumen cannula inserted into the SVC via the right internal jugular vein. Visualization of the guidewire within the correct vessel is paramount to safe cannula insertion.

Attention should be paid to properly visualizing the guidewires. The mid-esophageal bicaval and modified bicaval views with TEE provide excellent visualization of the IVC, SVC, TV and RA [28]. The guidewire should be seen in both cavae to confirm that it has not passed through the TV and into the RV, across an atrial septal defect, or into the coronary sinus. During the repeated dilatation of the skin and subcutaneous tissues, and whilst threading the cannula over the guidewire, it is necessary to maintain visualization of the guidewire to identify any secondary migration [29]. Attention should also be paid to monitoring any new or increasing pericardial collection [30].

For optimal drainage, the cannula tip should be located in the RA just beyond the caval-atrial junction (Additional files 1 and 2). If the cannula is not advanced far enough, there is an increased risk of the tip pressing against the wall of the IVC (Additional file 3). If it is too far into the RA, there is a risk of damage to structures, such as the interatrial septum (IAS; Additional file 4) or TV (Additional file 5). It will also increase the possibility of recirculation if the venous drainage cannula is too close to the reinjection one [31].

**Fig. 2:**
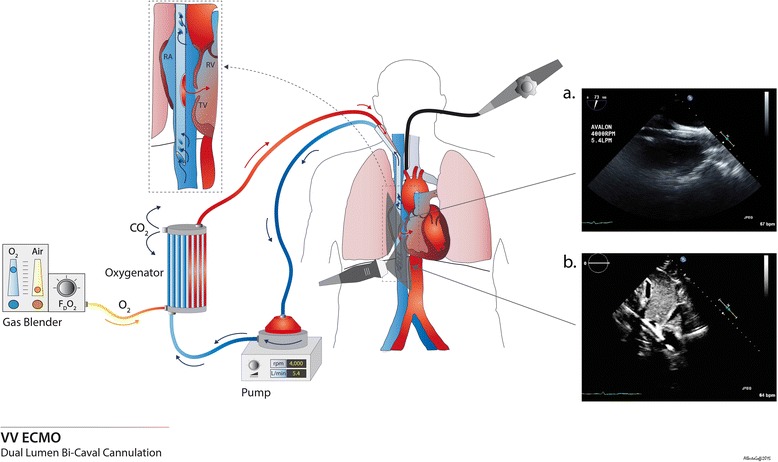
Bicaval dual-lumen cannula for venovenous extracorporeal membrane oxygenation (VV ECMO; Avalon Elite®) and corresponding echocardiographic views. This picture depicts a bicaval dual-lumen cannula, inserted via the internal jugular vein. The drainage holes are located in the superior vena cava and inferior vena cava (IVC), and the reinjection hole is facing the tricuspid valve (TV). **a** Mid-esophageal bicaval view showing the cannula within the right atrium (RA) (transesophageal echocardiography). **b** Transthoracic subcostal view showing the cannula in the RA; the tip of the cannula is located in the IVC. The reinjection hole is visible, oriented towards the tricuspid valve. RV, right ventricle

The Avalon Elite® (Maquet, Rastatt, Germany) bicaval dual-lumen cannula is designed to drain blood from both the IVC and SVC, and return oxygenated blood into the RA, with the flow directed towards the TV (Fig. 2). Its advantages are single site cannulation and a decreased propensity for recirculation [31]. Echocardiography provides excellent views of the appropriate position of the different portions of the cannula (Additional files 6 and 7). Correct positioning of the cannula tip in the IVC must be confirmed to avoid cannulation of one of the hepatic veins (Additional files 8, 9, and 10). The utilization of color flow Doppler can demonstrate the direction of the returned blood towards the TV, and not the IAS or into the hepatic veins (Additional files 11, 12 and 13) [32, 33].

As with VV ECMO, in VA ECMO blood is drained from the venous system, but returned into the arterial system after oxygenation and CO_2_ removal. In peripheral VA ECMO the venous drainage is similar to VV ECMO, with the drainage cannula positioned at the IVC-RA junction. The return cannula is usually inserted through the femoral artery, with the tip sitting in the iliac artery or distal aorta. Aortic ultrasound can be used to visualize the guidewire within the upper abdominal aorta, to avoid malposition of the arterial cannula within a branch vessel. In central VA ECMO the reinjection cannula is typically inserted directly into the patient’s ascending aorta.

Few studies in the pediatric literature have reported the added value of echocardiography to guide insertion and correct placement of ECMO [34, 35]. Intra-operative chest X-rays have failed to demonstrate malposition of the cannulae and did not provide any assessment of flows [34]. In another study, 13 % of patients required interventions, for example, modification of cannula position, increase in ECMO flows or upsize of the oxygenator, that were not detected on chest X-ray [36]. Some authors have described the use of contrast echocardiography to help guide more accurately the direction of the returned blood flow towards the TV in VV ECMO [32, 37, 38]. With the growing amount of patients supported with ECMO, an increasing number of reports describe the additional information provided by echocardiographic guidance of ECMO, with or without the use of concomitant fluoroscopy [39]. Use of echocardiography certainly does not prevent the occurrence of complications, but seems to be a valuable adjunct to other imaging methods. Equipoise still persists, some authors advocating the use of both fluoroscopy and echocardiography [40, 41].

## Patient monitoring during ECMO

### Venoarterial ECMO

Ideally a daily echocardiogram should be performed whilst the patient is on ECMO, since other methods to assess cardiac output may be unreliable (Table 2). Indeed, cardiac output measured with the thermodilution technique may be overestimated, as the blood is being suctioned from the RA. In addition, the pulse contour analysis method may be limited by the absence of pulsatility.

**Table 2:** Echocardiographic parameters on ECMO

	Venovenous ECMO	Venoarterial ECMO
Monitoring on ECMO	Biventricular size and function	Biventricular size and function
	Biatrial size and volume	Biatrial size and volume
	Follow up of any pre-existing pathology	Follow up of any pre-existing pathology
	Cannula position	Mitral/aortic regurgitation
	Pericardial effusion	Opening of aortic valve
	IVC size and collapsibility	Intracavitary spontaneous echo contrast/intracavitary thrombus
		Aortic thrombus
		Cannula position
		Pericardial effusion
		IVC size and collapsibility
Weaning from ECMO: measurements at baseline and with stepwise decrement on flows	LVEF	LVEF
	RV size and function (TAPSE, FAC, S at tricuspid annulus)	LVOT VTI
	Paradoxical septum	S wave at lateral annulus
	TR and RVSP	RV size and function
		TR and RVSP

*ECMO* extracorporeal membrane oxygenation, *FAC* fractional area change, *IVC* inferior vena cava, *LVEF* left ventricular ejection fraction, *LVOT VTI* left ventricular outflow tract velocity time integral, *RV* right ventricle, *RVSP* right ventricular systolic pressure, *TAPSE* tricuspid annular plane systolic excursion, *TR* tricuspid regurgitation

One important use of serial echocardiograms is to monitor cardiac chamber size to ensure adequate emptying of the ventricles. In addition, aortic valve opening is paramount. In peripheral VA ECMO, the retrograde aortic blood flow competes with the stroke volume ejected from the LV. A closed aortic valve will ultimately lead to LV distention and thrombus formation (Additional files 14 and 15). Intra-cavitary thrombus and those present within the aorta have been reported and diagnosed with the aid of echocardiography (Additional files 16, 17, 18, and 19) [42–46]. Presence of spontaneous echo contrast is indicative of blood stasis and suggests that the patient might be at higher risk of thrombus formation. Additionally, increased afterload may worsen pre-existing aortic regurgitation and LV distension, potentially hindering recovery by inducing subendocardial ischemia, increased myocardial oxygen consumption and subsequent pulmonary edema. The bronchial circulation and aortopulmonary collateral vessels can also return a significant amount of blood to the heart, contributing to further chamber dilatation. In cases of severe LV and LA dilatation, with associated pulmonary edema, some authors have reported a rapid resolution and improvement after LV decompression. However, the exact indications and timing are not well defined [47, 48].

Several methods have been described for LV decompression: a surgical approach with a minimally invasive thoracotomy; percutaneous approaches via the pulmonary artery or aortic valve; or through a septostomy [49, 50]. A more novel approach is with the use of an Impella® device (Abiomed, Danvers, MD, USA), a percutaneously inserted microaxial pump (Additional file 20) [51]. Generally the percutaneous approaches have been guided with fluoroscopy, but there are some reports describing the use of echocardiography to guide the procedure, and subsequently follow the transatrial gradients along with LA and LV dimensions [48, 49, 52–54]. LA decompression seems to be associated with a significant improvement of LV function, as observed on echocardiogram (Additional files 21, 22, 23, and 24) [48, 52]. Whether this improvement is linked to better outcomes needs to be further investigated.

Serial monitoring of biventricular function enables earlier detection of recovery, which may be assessed during echocardiography by modulating the ECMO flows. If recovery is present, increased biventricular contractility without severe RV dilatation, with reduced ECMO support (for example, 1 to 2 L/min blood flow), should be observed on echocardiography.

### Venovenous ECMO

There are few reports of echocardiographic monitoring of cardiac function during VV ECMO (Table 2) [5, 55, 56]. Nevertheless, echocardiography can help the physician to determine the cause of inadequate flows, which are not uncommon during ECMO support. Evidence of volume overload in ARDS patients typically prompts restrictive fluid therapy and/or diuresis, sometimes at the expense of inadequate ECMO flows [57–59]. Signs of hypovolemia or collapse of the IVC around the cannula may give an indication as to how fluid management (for example, aggressive diuresis) should be handled. However, reduced ECMO flows may be caused by cannula displacement, particularly the bicaval dual-lumen cannula, leading to significant hypoxemia [60]. Appropriate cannula position can be promptly confirmed and readjusted with TTE or TEE guidance, if needed [33].

On VV ECMO, recirculation occurs when the tips of the cannulae are located too close together, resulting in further patient hypoxemia. Cannulae may be repositioned as necessary [11]. On VV ECMO, cardiac function assessment should be assessed as clinically indicated [5].

### Common considerations for venovenous and venoarterial ECMO

As previously mentioned, limited flows during ECMO support are frequent both on VV and VA ECMO. Although this is usually related to patient positioning or hypovolemia, it is important to exclude the presence of an intra-cannula thrombus (Additional file 25). This is achieved by assessing the blood flow at the orifice of the drainage cannula with pulse wave and color flow Doppler [61].

The use of systemic anticoagulation makes patients on ECMO more susceptible to bleeding and potential pericardial collections. However, even in the presence of a pericardial effusion, the clinical and echocardiographic diagnosis of tamponade remains challenging, especially on VA ECMO, as the modification of RA and RV pressures may preclude appropriate analysis of chamber collapse and of its clinical significance.

## Weaning from ECMO

To our knowledge, there are currently no data on the utility of evaluating RV function by echocardiography during weaning from VV ECMO support. However, acute hypercapnia may precipitate pulmonary hypertension, reopening a PFO and leading to acute RV failure.

Most of the data pertaining to weaning derive from VA ECMO, although weaning strategies are highly center dependent and without well-defined standard operating procedures [62, 63]. Weaning from extracorporeal life support is considered when there are signs of cardiac recovery. These include improved ventricular contractility and consistent opening of the aortic valve. In clinical practice, the ECMO flows are decreased under close hemodynamic and echocardiographic monitoring. Usually flows are decreased to approximately 1 to 1.5 L/min, with a potential increase in the risk of circuit thrombus formation. Until ECMO flows are completely interrupted, it is difficult to predict with certainty that weaning will be successful, as acute RV dysfunction can be masked even with minimal ECMO flows. Feasibility of using a disposable TEE probe has been studied in an attempt to define a weaning protocol, allowing continuous monitoring of echocardiographic parameters whilst decreasing the ECMO flows. Whether this would have any clinical impact still remains to be proven [64].

Attempts have been made to delineate parameters beyond the usual EF and ventricular size, to predict successful weaning from VA ECMO [65]. Whilst decreasing the ECMO flow in a stepwise fashion, the presence of an aortic velocity-time integral greater than 10 cm, at minimal ECMO support, was predictive of successful weaning. In addition to the velocity-time integral, an ejection fraction above 20 to 25 % and a systolic S wave velocity (Sa) greater than 6 cm/s at the lateral annulus of the mitral valve (tissue Doppler imaging), were associated with successful weaning from ECMO. In another study, an increase in strain and strain rate of 20 % at minimal ECMO flows, compared to baseline, with a concomitant increase of EF, could predict successful weaning [66]. The strain and strain rate remained unchanged in patients that could not be successfully weaned. A case report describes the analysis of descending aortic blood flow to follow up recovery of cardiac function, showing a progressive increase of anterograde flow [67].

## Post-ECMO phase

One of the main concerns after decannulation is the presence of thrombus or obstruction that may have not been identified when the cannulae were *in situ* (Additional file 26) [19]. Zreik et al. [68] reported an incidence of SVC obstruction in 7 of 50 children post-ECMO removal.

## Conclusion

Echocardiography plays a crucial role at every step of ECMO support. At the time of consideration, it can confirm the diagnosis and help in defining the choice between VV and VA ECMO. Although, it is unlikely that utilization of echocardiography by itself will directly improve the outcome of patients supported on ECMO, echocardiography may help to reduce complications and guide clinicians in the daily management of these complex patients [8]. It provides guidance at the time of cannulation. Once the patient is on ECMO support, it procures valuable information pertaining to recovery and possible complications. Finally, it is essential during the weaning phase for VA ECMO.

There are currently no guidelines with respect to the optimal monitoring of patients on ECMO. Our review has emphasized specific echocardiographic parameters that might be important to follow over the course of ECMO support. Many questions remain unaddressed and require further investigation. For instance, the benefits of echocardiography during insertion of ECMO and during maintenance of VV ECMO support and weaning need to be better defined. Moreover, guidelines for standardized echocardiographic measurements during weaning from VA ECMO would help to guide physicians caring for these patients. Finally, the utility of monitoring RV function during VV ECMO support requires further evaluation.

In conclusion, echocardiography is an essential monitoring tool in the care of patients on ECMO. Further investigations and guidelines should help delineate its use for patients on ECMO support.

## Additional files

Additional file 1: Image S1. Inferior vena cava/right atrium junction (TEE). Three-dimensional imaging of the cannula in IVC and RA. (PNG 409 kb) Additonal file 2: Video S1. Patient on VV ECMO. Subcostal view (TTE) showing the drainage cannula within the IVC. Cannula properly positioned with the tip in the RA just beyond the IVC/RA junction. (MP4 4761 kb) Additional file 3: Video S2. Patient on VV ECMO. Subcostal view (TTE). The drainage cannula is not advanced far enough. The tip of the cannula is abutting the posterior wall of the IVC. (MP4 5859 kb) Additional file 4: Video S3. Patient on VV ECMO. Bicaval view (TEE). Cannula located within the RA with the tip abutting against the IAS. The cannula is progressively withdrawn under echocardiography guidance. (MP4 2934 kb) Additional file 5: Video S4. Patient on VV ECMO. Apical four-chamber view (TTE). Avalon Elite® cannula visualized in the RA and going through the TV. (MP4 2390 kb) Additional file 6: Videos S5. Cannulation for VV ECMO. Bicaval views (TEE) showing the progressive advancement of an Avalon® cannula, through RA and IVC. (MP4 828 kb) Additional file 7: Videos S6. Cannulation for VV ECMO. Bicaval views (TEE) showing the progressive advancement of an Avalon® cannula, through RA and IVC. (MP4 3777 kb) Additional file 8: Image S2. IVC subcostal view. Correctly positioned Avalon® (TTE). The tip of the cannula is in the IVC, the reinjection orifice is directed towards the tricuspid valve. (PNG 281 kb) Additional file 9: Image S3. Abdominal ultrasound showing an Avalon® in one of the hepatic veins. (PNG 256 kb) Additional file 10: Video S7. Patient on VV ECMO. Subcostal view (TTE). Avalon® visualized through the RA with the tip properly positioned in the IVC. Note that the reinjection orifice is also visible and properly directed toward the TV. (MP4 3744 kb) Additional file 11: Video S8. Same patient as in Additional file 10. VV ECMO Avalon®. Subcostal view with color flow Doppler with the reinjection flow towards the TV. (MP4 2304 kb) Additional file 12: Video S9. Patient on VV ECMO with an Avalon® cannula. Bicaval view (TEE) with color flow Doppler showing the flow inappropriately directed towards the IAS. (MP4 2304 kb) Additional file 13: Video S10. Patient on VV ECMO with an Avalon® cannula with reinjection flow in hepatic veins. Subcostal view (TTE). (MP4 2235 kb) Additional file 14: Image S4. Apical four-chamber view of a patient on VA ECMO. Apical LV thrombus (TTE). (PNG 535 kb) Additional file 15: Video S11. Patient on VA ECMO. Mid-esophageal three-chamber view (TEE) showing a closed aortic valve and spontaneous echo contrast in the LV. (MOV 1239 kb) Additional file 16: Video S12. Patient on VA ECMO. Parasternal long axis view (TTE). Severely decreased LV systolic function with LV thrombus. (MP4 2693 kb) Additional file 17: Video S13. Same patient as in Additional file 16. Parasternal short axis view (TTE) showing two LV thrombi (one at the junction of the infero-septal and inferior wall, the other at the junction of the antero-septal and anterior wall). (MP4 4585 kb) Additional file 18: Video S14. Patient on VA ECMO. Mid-esophageal RV inflow-outflow view (TEE) showing a RV thrombus extending into the main PA. (MP4 5868 kb) Additional file 19: Video S15. Patient on VA ECMO. Mid-esophageal X plane (two-chamber and three-chamber view; TEE) depicting LA thrombus. (MP4 5874 kb) Additional file 20: Video S16. Patient on VA ECMO. LV decompression with an Impella® through the aortic valve. Note that the tip of the Impella® is not positioned properly as it is abutting the mitral subvalvular apparatus. (MP4 2887 kb) Additional file 21: Image S5. Parasternal short axis view of a patient on VA ECMO who underwent a septostomy for LA decompression (TTE). LA vent cannula seen in the LA and through IAS. (PNG 537 kb) Additional file 22: Video S17. Patient on VA ECMO. Parasternal long axis view (TTE): severely impaired LV function and dilation without opening of the aortic valve. (MP4 2406 kb) Additional file 23: Video S18. Patient on VA ECMO. Parasternal long axis of the same patient as in Additional file 22 after LA decompression via septostomy. The LA vent is visualized in the LA. The aortic valve is now opening at every beat. (MOV 1320 kb) Additional file 24: Video S19. Patient on VA ECMO. Parasternal short axis at the level of the aortic valve of the same patient as in Additional files 22 and 23. LA vent cannula seen is the LA through the IAS. (MOV 1249 kb) Additional file 25: Video S20. Patient on VA ECMO. Apical four-chamber view (TTE). LA decompression. LA vent seen in the LA with mobile mass at the tip, consistent with a thrombus. This study was performed with lower ECMO flows to better assess recovery. (MOV 1258 kb) Additional file 26: Video S21. IVC subcostal view (TTE). IVC thrombus post ECMO cannula removal. (MP4 2407 kb)

## Abbreviations

ARDS: acute respiratory distress syndrome
CO_2_: carbon dioxide
ECMO: extracorporeal membrane oxygenation
EF: ejection fraction
IAS: interatrial septum
IVC: inferior vena cava
LA: left atrium
LV: left ventricle
PA: pulmonary artery
PFO: patent foramen ovale
RA: right atrium
RV: right ventricle
SVC: superior vena cava
TEE: transesophageal echocardiography
TR: tricuspid regurgitation
TTE: transthoracic echocardiography
TV: tricuspid valve
VA: venoarterial
VV: venovenous
