# Erratum to: Echocardiography for adult patients supported with extracorporeal membrane oxygenation

**DOI:** 10.1186/s13054-016-1214-8

**Published:** 2016-02-08

**Authors:** Ghislaine Douflé, Andrew Roscoe, Filio Billia, Eddy Fan

**Affiliations:** 1 Interdepartmental Division of Critical Care Medicine, University of Toronto, Toronto, ON M5G 2N2 Canada; 2 Extracorporeal Life Support (ECLS) Program, Toronto General Hospital, Toronto, ON M5G 2N2 Canada; 3 Department of Anaesthesia & ICU, Papworth Hospital, Cambridge, CB23 3RE UK; 4 Peter Munk Cardiac Centre, University Health Network, Toronto, ON M5G 2N2 Canada

Upon publication of this article [1] it has been found that the additional files published out of order by mistake. This has now been corrected.

In addition we updated a minor error in the legend for additional file 24:

‘Additional file 24. Video S19. Patient on VA ECMO. Parasternal short axis at the level of the aortic valve of the same patient as in Additional files 22 and 23. LA vent cannula seen is the LA through the IAS.’

The legend is now correctly referring to additional files 22 and 23, rather than 22 and 18 as before.
