# Supplementary figures and images for: Echocardiography for adult patients supported with extracorporeal membrane oxygenation
Source: Crit Care. 2015 Oct 2;19:326. doi: 10.1186/s13054-015-1042-2 (PMC4591622; doi:10.1186/s13054-015-1042-2)

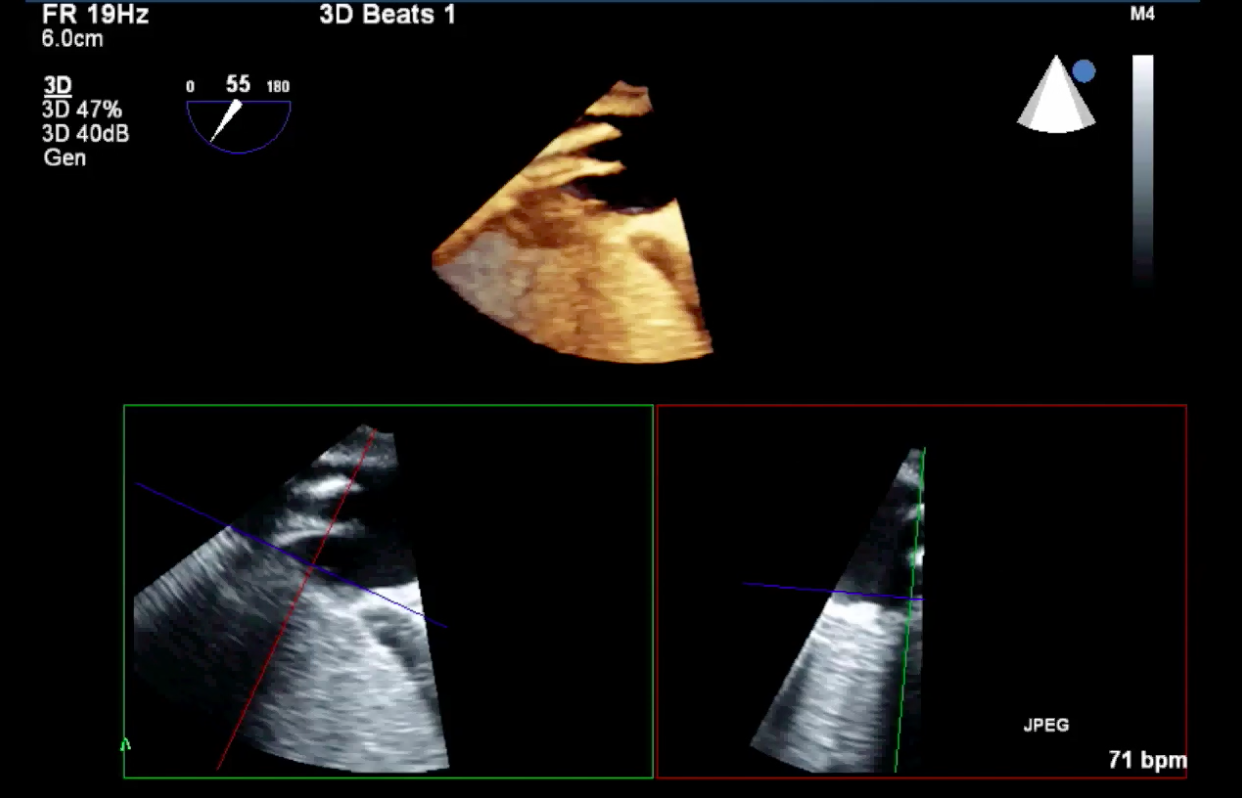

Supplement: Additional file 1: Image S1. — Inferior vena cava/right atrium junction (TEE). Three-dimensional imaging of the cannula in IVC and RA. (PNG 409 kb) [file 13054_2015_1042_MOESM1_ESM.png]

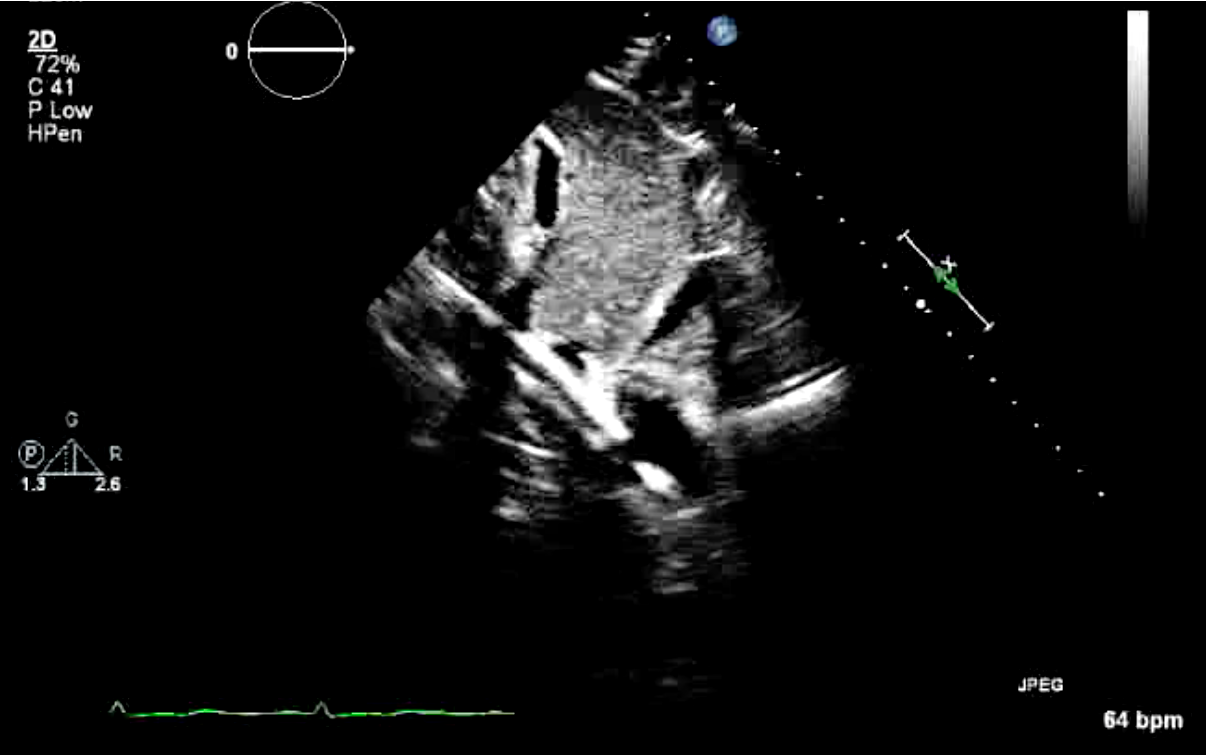

Supplement: Additional file 8: Image S2. — IVC subcostal view. Correctly positioned Avalon® (TTE). The tip of the cannula is in the IVC, the reinjection orifice is directed towards the tricuspid valve. (PNG 281 kb) [file 13054_2015_1042_MOESM8_ESM.png]

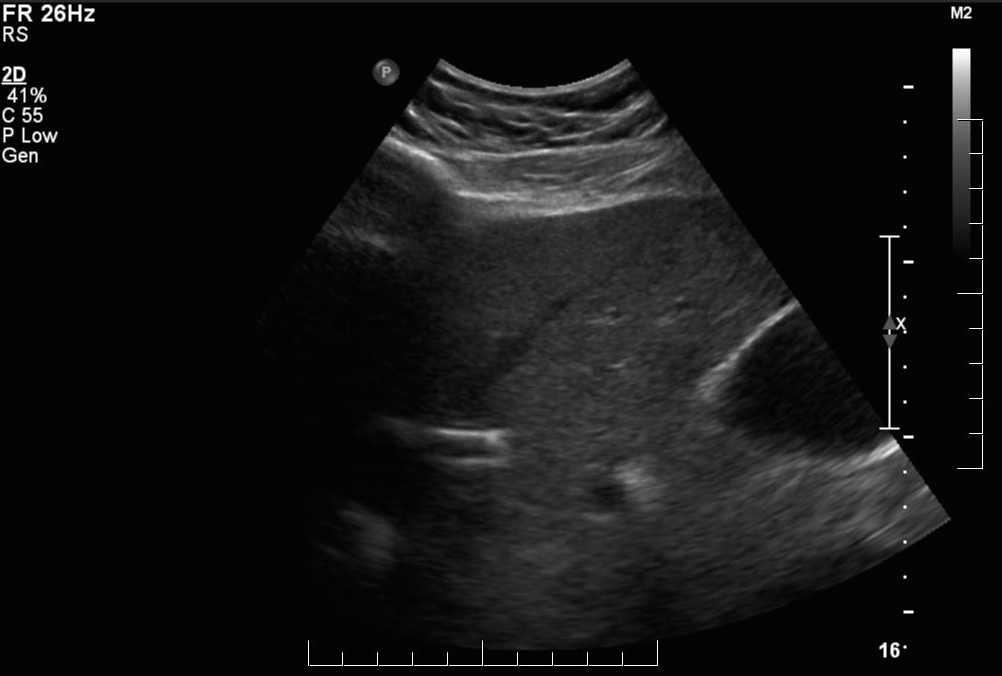

Supplement: Additional file 9: Image S3. — Abdominal ultrasound showing an Avalon® in one of the hepatic veins. (PNG 256 kb) [file 13054_2015_1042_MOESM9_ESM.png]

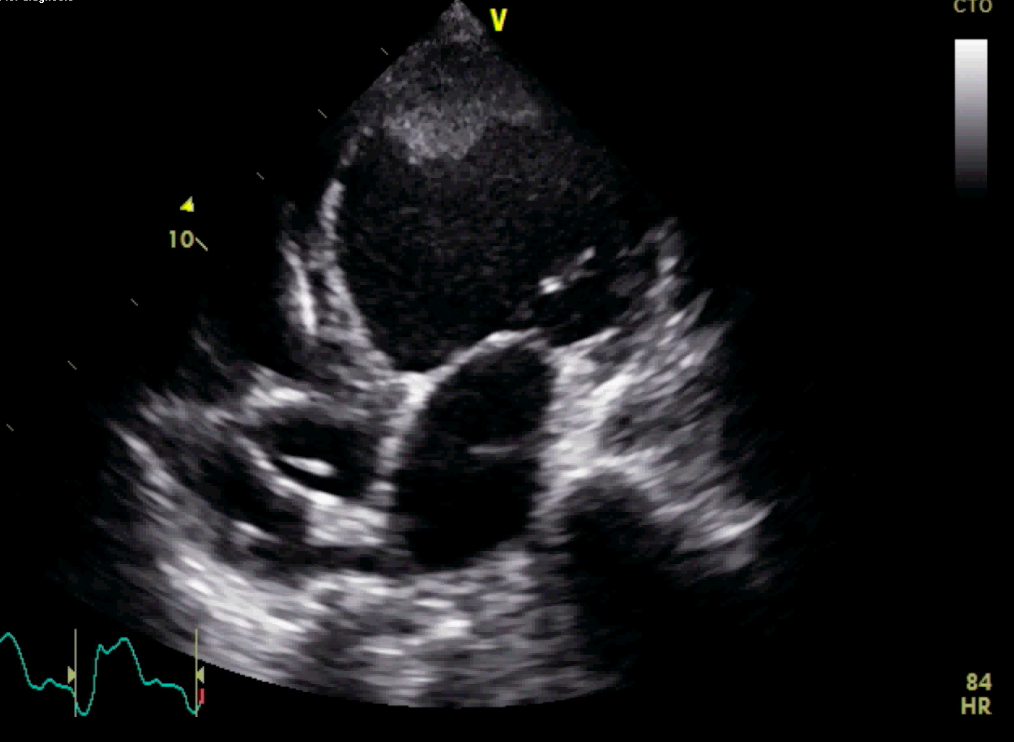

Supplement: Additional file 14: Image S4. — Apical four-chamber view of a patient on VA ECMO. Apical LV thrombus (TTE). (PNG 535 kb) [file 13054_2015_1042_MOESM14_ESM.png]

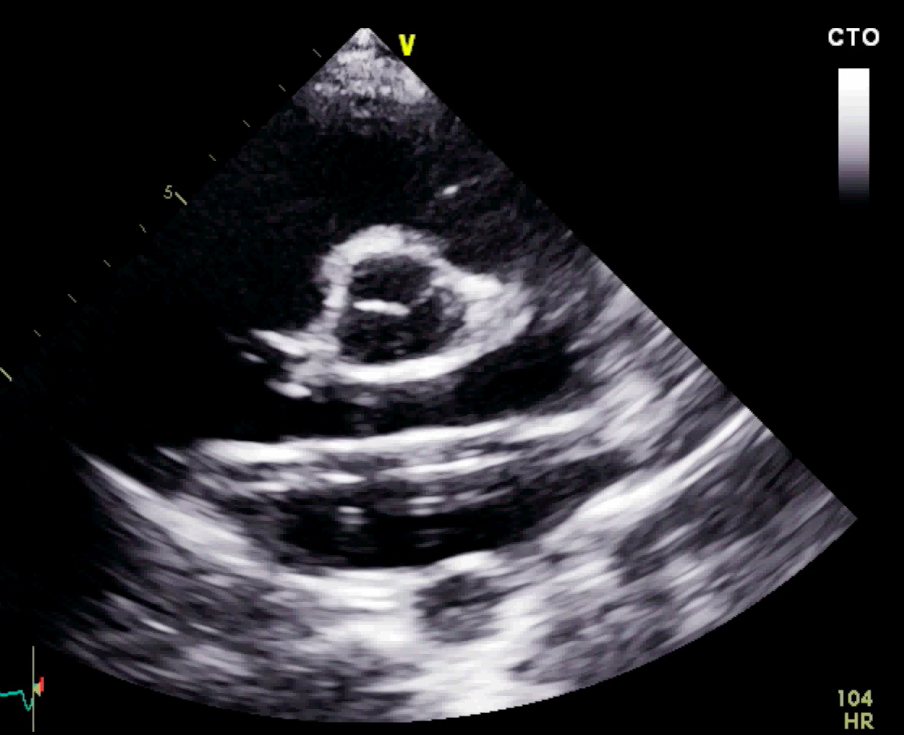

Supplement: Additional file 21: Image S5. — Parasternal short axis view of a patient on VA ECMO who underwent a septostomy for LA decompression (TTE). LA vent cannula seen in the LA and through IAS. (PNG 537 kb) [file 13054_2015_1042_MOESM21_ESM.png]
